# Respiratory Diseases

**Published:** 1903-11-07

**Authors:** 


(*To be continued.*)

### Respiratory Diseases.

**Treatment and Care of Consumptives at their Homes.**[1]—S. A. Knopf contributes a valuable article on this subject. He deals with the question of disposal of sputum, and the necessity of care on the part of the patient in coughing and sneezing so that infection may not be transmitted in this way, and condemns the practice of coughing whenever the throat tickles. He warns us against the evils of too much clothing, especially of the chest protector type, holding that a patient's usual underwear, if rational, is the best for the individual case. For women, a dress made in one piece is desirable, so that the waist is not fixed by being made to support the lower part. The diet should be a mixed one—meat, fish, fresh vegetables and fruit, fresh milk and easily digested fats (butter is specially good), thick soups, raw chopped or "scraped" beef; no alcohol unless specially ordered. The food should be well chewed, and liquid taken in sips with the food. Too much sweet food is to be avoided, so also is fried food. The teeth should be carefully attended to. Massage is of value, and should be practised on a suitable table rather than on the bed, for the convenience of the operator. An illustration is given in the original article of the kind of outdoor shelter that may be easily built on to the patient's residence. A well-ventilated room with south or south-west aspect should be used. For rest, a chair with a sloping back, and a long seat, with a hump to support the patient's bent knees is the best form, but an ordinary deck chair will serve. The beach chair so popular at American seaside resorts is very good too, as its high back and wings afford protection from wind. Exercise should not be permitted while there is any fever, and should be avoided if the patient is at all tired, nor should it ever be so pushed as to produce fatigue, or a rise of temperature. The thermometer is a valuable guide to the physician in determining the proper amount of exercise. The author refers briefly to his breathing exercises, on which he has repeatedly written, and goes on to give details as to sun-baths. The patient must be unclothed gradually, and the amount of exposure must not be so great as to produce skin troubles, headache, or discomfort in susceptible persons. Pyrexia is a contra-indication. Hydrotherapy is of the greatest importance, and if no better bath can be arranged, a few pitchers of water should be rapidly poured over the patient's head and shoulders as he sits astride a chair bending his face over the back. Friction with a towel must follow. The best time for the bath is the early morning after a preliminary light breakfast and a short walk, the more substantial breakfast following.

**Decrease of Tuberculosis in New York.**[2]—In 1886 the death-rate from all forms of tuberculosis was 4·5 per thousand; it was 2·68 during the past year. An attempt is being made, however, to weaken the law regulating the construction of tenement houses, and as the decrease is regarded as being largely due to the greatly improved dwellings of the masses, the medical fraternity is protesting against this attempt.[3]

**Etiology of Tuberculosis.**—Gordon[4] concludes that though wind in itself is harmless to phthisical patients, the wet winds prevalent in the south-west of England, and in fact over England generally, are a factor in the production of tuberculosis apart from any tendency they may have to induce indoor life. Aufrecht[5] has made an extensive investigation with a view to determining the mode of entrance of the tubercle bacillus into the lungs and tissues generally, and finds that in many cases the tonsils are the portal. The cervical glands become tuberculous, and from these the bacilli gain access to the venous circulation, and reach the pulmonary capillaries *via* the right heart. They lodge especially at the apices where movement is usually least, and where, he thinks, injury may sometimes be produced by too violent inspiration. J. Wier[6] records a case of a man in whom a bruise on the ribs was followed within a month by lassitude, night-sweats, loss of weight and of appetite. Two months later there were signs of consolidation at the site of injury and also at the apex, and after another two months the diagnosis of phthisis was confirmed by finding the bacilli in the sputum, and by the presence of cough and hæmoptysis. Curiously enough, the patient's sister was said to have died of phthisis brought on by injury to the chest produced by a catapult shot.

**Ventilation of Railway Carriages.**—Now that railway journeys frequently last for several hours without the carriage door being opened, it has become a matter of importance that something should be done to improve the ventilation. The *Lancet*[7] has drawn attention to this weak point on our railways. The "ventilating" shutter is practically useless, and as much as 10 parts per 10,000 of $CO_2$ is commonly found in closed compartments.

**Therapeutic Treatment of Phthisis.** — Sanchez Herrero (Madrid)[8] uses cinnamate of sodium in doses 10 to 20 times greater than are used by Landerer. Commencing with a 3 cc. dose of a 4 per cent. solution, he increases it to 5 cc., and continues the injection of this amount daily; if no improvement occurs in ten days he increases the dose to 6, or 8 cc. In one case he raised it to 17 cc. He speaks with great satisfaction of the results, and says that the injections are not painful. Lovell Drage[9] also records a case of pulmonary tuberculosis, in an early acute stage, which recovered completely under injections of a 10 per cent. glycerine solution of the salt in doses of 30 minims at intervals of six or seven days. Subcutaneous injections of yolk of egg[10] have been employed in America without harmful results, as a means of feeding tuberculous patients who could not digest their food sufficiently. It is mixed with an equal part of saline solution, and of this fluid mixture as much as an ounce can be injected subcutaneously without producing local irritation. The lecithin has a higher nutritive value thus given, because it is not broken down and wasted to so great an extent as when it is acted on by the digestive juices before absorption. Reisman[11] recommends the use of hedonal in doses of 30 grains to relieve insomnia in phthisis. It acts, he says, as a pure hypnotic, with no unpleasant after-effects and no tendency to produce a habit.

**Oral and Rec al Temperatures.**[12]—Burton Fanning and Champion show that there are very marked differences between the oral temperature under certain circumstances, and the true body temperature as indicated in the rectum or elsewhere. Breathing cool air, mouth-breathing and rapid breathing during exercise, or cold air acting through the cheeks, considerably lower the oral temperature and it takes sometimes as much as half an hour in a warm still atmosphere to obtain a maximum reading in the mouth. A patient in bed, however, gives an oral reading which corresponds fairly closely to the rectal temperature. Careful observations show that the temperature of healthy persons rises far higher than is commonly supposed with exercise. After half an hour's brisk walking, a rise in the rectal temperature to 101·0° or a little more, is usual, and this is maintained until on taking rest there is a gradual fall to the accepted normal.

**Drowning.**[13]—The Royal Medical and Chirurgical Society, at a meeting on May 26th, discussed means of resuscitating the apparently drowned. Professor Schafer read an account of his experiments on dogs and on man. The results supported Sylvester's method, giving preference to the employment of the prone position while raising the arms, and pressing on the back at the same time that the arms are pressed against the sides. His experiments on dogs showed the danger of using too much pressure, as in one or two cases, the liver, always greatly engorged in asphyxia, had been ruptured by the violence of the efforts to make the lungs act. This accident has been noted in one or two cases of chloroform asphyxia where artificial respiration was vigorously pushed. In the dogs, though the case might have ended fatally, it was found that as much as half a litre of water was sometimes absorbed from the lungs if the water was fresh, but if salt water invaded the lung, the contrary process took place, water was poured out by the tissues into the alveoli.

1 Med. Rec., Feb. 21, 1903. 2 Ibid. 3 Med. Rec., March 7. 4 Brit. Med. Jour., May 23. 5 Med. Rec., June 27. 6 Brit Med. Jour., May 23. 7 Lancet, July 11. 8 Med. Rec., May 30. 9 Lancet, May 23. 10 Amer. Jour. Med. Sci., March. 11 Clin. Excerpts, Jan. 12 Lancet, March 28. 13 Lancet, May 30.

